# Circulating Exosomal miRNAs Signal Circadian Misalignment to Peripheral Metabolic Tissues

**DOI:** 10.3390/ijms21176396

**Published:** 2020-09-03

**Authors:** Abdelnaby Khalyfa, Shobhan Gaddameedhi, Elena Crooks, Chunling Zhang, Yan Li, Zhuanhong Qiao, Wojciech Trzepizur, Steve A. Kay, Jorge Andrade, Brieann C. Satterfield, Devon A. Hansen, Leila Kheirandish-Gozal, Hans P. A. Van Dongen, David Gozal

**Affiliations:** 1 Department of Child Health, Child Health Research Institute, University of Missouri School of Medicine, Columbia, MO 65201, USA; khalyfaa@missouri.edu (A.K.); qiao_zhuanhong@hotmail.com (Z.Q.); gozall@health.missouri.edu (L.K.-G.); 2 Department of Biological Sciences, Center for Human Health and the Environment, North Carolina State University, Raleigh, NC 27606, USA; sgaddam4@ncsu.edu; 3 Sleep and Performance Research Center, Washington State University, Spokane, WA 99202, USA; ecrooks@ewu.edu (E.C.); satterfield@wsu.edu (B.C.S.); devon.hansen@wsu.edu (D.A.H.); hvd@wsu.edu (H.P.A.V.D.); 4 Department of Physical Therapy, Eastern Washington University, Spokane, WA 99202, USA; 5 Center for Research Informatics, Biological Sciences Division, Pritzker School of Medicine, The University of Chicago, Chicago, IL 60637, USA; zhangch@upstate.edu (C.Z.); yli22@bsd.uchicago.edu (Y.L.); andrade.jorge@gmail.com (J.A.); 6 Département de Pneumologie, INSERM UMR 1063 SOPAM, Centre Hospitalier Universitaire, 49100 Angers, France; wotrzepizur@chu-angers.fr; 7 Department of Neurology, Keck School of Medicine, University of Southern California, Los Angeles, CA 90007, USA; stevekay@usc.edu; 8 Elson S. Floyd College of Medicine, Washington State University Health Sciences Spokane, Spokane, WA 99202, USA

**Keywords:** Bmal1-dLuc reporter assay, circadian rhythm, clock genes, constant routine, exosomes, extracellular vesicles, hsa-mir-3614-5p, insulin resistance, night shift work, peripheral oscillators

## Abstract

Night shift work increases risk of metabolic disorders, particularly obesity and insulin resistance. While the underlying mechanisms are unknown, evidence points to misalignment of peripheral oscillators causing metabolic disturbances. A pathway conveying such misalignment may involve exosome-based intercellular communication. Fourteen volunteers were assigned to a simulated day shift (DS) or night shift (NS) condition. After 3 days on the simulated shift schedule, blood samples were collected during a 24-h constant routine protocol. Exosomes were isolated from the plasma samples from each of the blood draws. Exosomes were added to naïve differentiated adipocytes, and insulin-induced pAkt/Akt expression changes were assessed. ChIP-Seq analyses for BMAL1 protein, mRNA microarrays and exosomal miRNA arrays combined with bioinformatics and functional effects of agomirs and antagomirs targeting miRNAs in NS and DS exosomal cargo were examined. Human adipocytes treated with exosomes from the NS condition showed altered Akt phosphorylation responses to insulin in comparison to those treated with exosomes from the DS condition. BMAL1 ChIP-Seq of exosome-treated adipocytes showed 42,037 binding sites in the DS condition and 5538 sites in the NS condition, with a large proportion of BMAL1 targets including genes encoding for metabolic regulators. A significant and restricted miRNA exosomal signature emerged after exposure to the NS condition. Among the exosomal miRNAs regulated differentially after 3 days of simulated NS versus DS, proof-of-concept validation of circadian misalignment signaling was demonstrated with hsa-mir-3614-5p. Exosomes from the NS condition markedly altered expression of key genes related to circadian rhythm in several cultured cell types, including adipocytes, myocytes, and hepatocytes, along with significant changes in 29 genes and downstream gene network interactions. Our results indicate that a simulated NS schedule leads to changes in exosomal cargo in the circulation. These changes promote reduction of insulin sensitivity of adipocytes in vitro and alter the expression of core clock genes in peripheral tissues. Circulating exosomal miRNAs may play an important role in metabolic dysfunction in NS workers by serving as messengers of circadian misalignment to peripheral tissues.

## 1. Introduction

**Figure 1:**
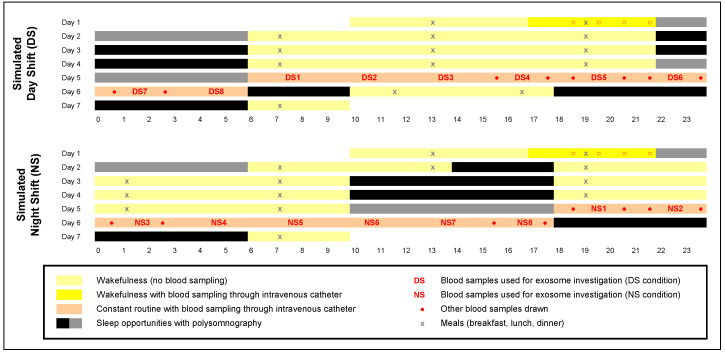
In-laboratory study consisting of a baseline period, 3 days on a simulated day shift (DS) schedule (top panel) or night shift (NS) schedule (bottom panel), 24 h constant routine (with hourly isocaloric snacks), and a recovery period. Blood samples were collected during the baseline period (dark yellow) and throughout the 24 h constant routine (salmon-colored). Blood samples were used for assessment of the dim light melatonin onset (Figure S1A) and targeted metabolomics (Figure S1B) [4] and for the investigation of exosomes (numbered samples DS1–DS8 and NS1–NS8; Figure 5). Sleep opportunities were measured with polysomnography (Figure S1C shows total sleep times for gray-marked sleep opportunities). Figure adapted from Skene and colleagues [4] with permission.

Human physiological homeostasis is subject to daily rhythms that are tightly controlled by the circadian clock. Shifted behavioral rhythms, such as those experienced during night shift (NS) work, lead to metabolic dysfunction and increased risk of obesity and type 2 diabetes mellitus [1,2,3]. The adverse health consequences of NS appear to be rooted in circadian misalignment [1] whereby shifted behavioral rhythms, including sleep/wake and feeding/fasting cycles, may cause misalignment between central and peripheral timekeepers. Consistent with this idea, we found that simulated NS work in the laboratory (Figure 1) induces profound misalignment between the central pacemaker in the SCN and metabolite rhythms that appear to reflect peripheral oscillators in the gut, liver, and pancreas [4]. Notably, this misalignment was observed under constant routine conditions—sustained wakefulness in constant ambient temperature and dim light, fixed semi-recumbent posture, and hourly isocaloric snacks [5]—which exposed endogenous circadian rhythms free of confounds from sleep/wake cycles or external influences (Figure S1).

Circadian oscillators are composed of auto-regulatory, transcriptional–translational feedback loops comprised of a core set of clock genes [6], which function to regulate circadian gene expression and their downstream targets. However, the processes governing central to peripheral clock interactions are unclear [7,8,9,10,11]. Understanding the genome-wide signaling of circadian gene expression is crucial to understanding the peripheral effects of night shift work. *BMAL1* (*ARNTL*), which is a critical circadian transcription factor that positively regulates other genes in their promoters, may be an important mediator of these effects [12,13].

Exosomes are ubiquitous extracellular vesicles (30–120 nm in size) that interact with target cells via multiple pathways [14,15] and regulate cell metabolism, proliferation, and differentiation [16,17,18]. Exosomes carry unique cargo containing proteins, lipids, DNA, messenger RNA (mRNA), and non-coding RNAs, and can transfer genetic information to recipient cells. Here, we propose that circulating exosomes may interact with *BMAL1* and operate as an underlying mechanism conveying signaling of circadian misalignment to peripheral tissues in the context of NS work. 

Indeed, exosomes play an important role in various biological processes, such as intercellular signaling, coagulation, inflammation, and cellular homeostasis. [19]. Exosomes can release their cargo to both neighboring and distal cells, serving important roles in intercellular communication [14,15,20,21,22,23]. This cargo includes miRNAs, which are 19–22 nucleotide-long, non-coding RNAs that function as negative regulators of translation. The miRNA cargo of exosomes exhibits high selectivity, and structural and functional stability [24,25]. Packaging of miRNAs within the exosomal lipid bilayers protects them from enzymatic degradation by body fluids, resulting in a relatively long and stable duration of expression [26,27].

Exosomal miRNAs delivered to target cells can affect biological pathways within these cells, resulting in altered cellular function and potentially causing the development of a pathological state [26,28]. Plasma-derived exosomes can interact with target tissues and cellular substrates and orchestrate the enrollment of inflammatory cells, e.g., to alter adipocyte metabolic pathways, thereby promoting the development of insulin resistance [29,30,31,32,33]. Exosomes secreted from skeletal muscle, visceral adipose tissue, and hepatocytes can transfer functional proteins and RNA species that regulate the metabolic function of adjacent cells as well as remote tissues [34,35]. The identification of mRNAs and miRNAs in exosomes and the ability of the transferred exosomal mRNA and miRNA to be translated in target cells is a major breakthrough in exosome biology [21]. 

Involved in many cellular processes [36,37], exosomal miRNAs play important roles in metabolic diseases, where they may be regarded as biomarkers and targets for correcting disturbances in metabolism [38,39]. It has been reported that exosomes shuttle nucleic acids to enter target cells, and these nucleic acids are translated into the encoded protein [20]. Furthermore, exosomal miRNAs can travel between cells and suppress the expression of target genes in recipient cells [14,22,23,40,41,42]. Although both exosomes and miRNAs have become the focus of intense research, little is known about exosome cargo regarding circadian rhythms, and more particularly in the context of NS work. 

We hypothesized that circadian misalignment is conveyed to peripheral tissues by exosomes, which transfer their cargo to acceptor target cells, which then alter their metabolic function, leading to increased insulin resistance. We investigated this by comparing the effects of three days of simulated NS work versus three days of simulated day shift (DS) work on homeostatic model assessment insulin resistance (HOMA-IR) values, miRNA profiling, systemic analyses based on chromatin immunoprecipitation sequencing (ChIP-Seq), and mRNA microarrays combined with bioinformatics to generate genome-wide profiles of target genes of the circadian clock gene, *BMAL1*. Additionally, we utilized agomir and antagomir approaches in naïve human adipocytes and applied a specific miRNA, hsa-mir-3614-5p, to provide proof of concept of the importance of exosome miRNA cargo as a signaling mechanism for circadian misalignment and insulin resistance. 

## 2. Results

### 2.1. Exosome Characterization

Negative stain electron microscopy of exosomes isolated from the blood samples taken during the 24-h constant routine (Figure 1) showed typical exosome morphology (Figure S3) consistent with previously published results [25]. Flow cytometry of isolated exosomes derived from the DS and NS conditions, using samples from early morning time points DS1 and NS5 matched for time of day (Figure 1), revealed the presence of tetraspanins, target/adhesion, and membrane transport and fusion markers, as anticipated in highly purified (>96%) exosome fractions (Figure S2). Quantification of isolated exosomes across the 24-h constant routine (Figure S4) showed no significant difference in the number of exosomes derived from the DS condition (grand average: 4.44 ± 0.34 10^8^/ml) versus the NS condition (grand average: 4.50 ± 0.41 10^8^/ml). 

Exosomes derived from the blood samples and labeled with Exo-Red (for RNAs), Exo-Green (for proteins), or PKH67 (for lipids) added to naïve differentiated human cell lines in vitro were effectively delivered to adipocytes (Figure S5), skeletal myocytes (Figure S6), and hepatocytes (Figure S7). Their contents were incorporated into these cells, where labeling with Exo-Red or Exo-Green showed that RNA and protein were localized to perinuclear and cytoplasmic regions, whereas labeling with PKH67 showed that lipids were restricted to the cell membrane. Cells exposed to control medium without exosomes did not show these effects.

### 2.2. Exosomal miRNA Cargo

**Figure 2:**
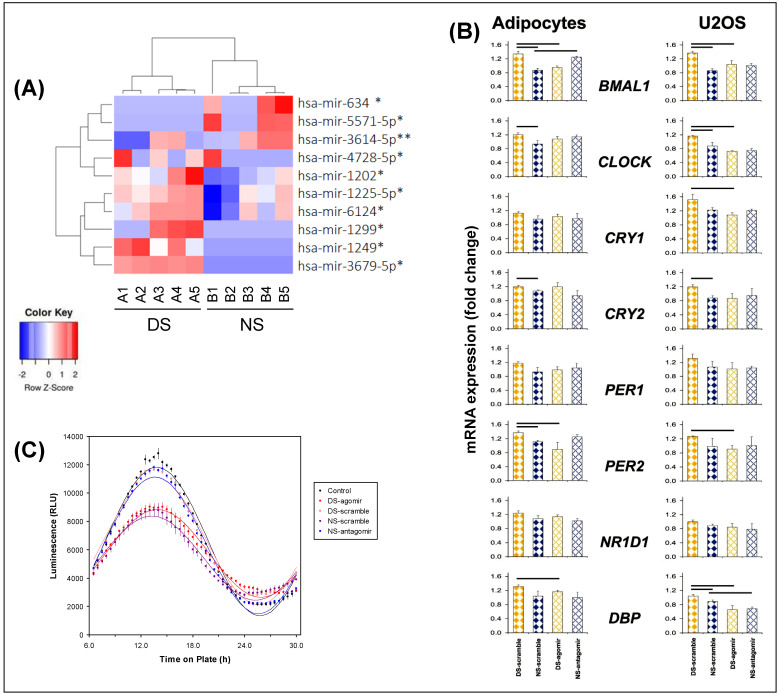
Profiling of plasma-derived exosome miRNAs. (**A**) Heatmap with clustering for miRNAs expressed differentially (red, increased miRNA abundance; blue, reduced miRNA abundance) at early morning time points DS1 versus NS5, matched for time of day (see Figure 1), for the top 10 up- or downregulated miRNAs (* *p* < 0.05, ** *p* < 0.01). Each column corresponds to a different subject exposed to either the DS condition (*n* = 5) or the NS condition (*n* = 5). Labels A1–A5 represent individuals in the DS condition; labels B1–B5 represent individuals in the NS condition. (**B**) Quantitative reverse transcription polymerase chain reaction (qRT-PCR) analysis of clock genes in adipocytes and U2OS cells treated with plasma-derived exosomes from subjects in the DS and NS conditions after transfection with hsa-mir-3614-5p scramble, agomir, and antagomir. Gene expression for each gene was normalized to DS or NS without exosomes. β-actin (*ACTB*) was used as housekeeping gene. Data represent averages of four independent replicates (error bars: SE); horizontal bars indicate significant difference (*p* < 0.05). (**C**) Luciferase reporter assay of Bmal1 rhythmicity in Bmal1-dLuc U2OS osteosarcoma cells treated with plasma-derived exosomes from early morning time points DS1 and NS5 (*n* = 7 per condition), matched for time of day, after transfection with miRNA-3614-5p scramble, agomir, or antagomir (or non-transfected controls). Total bioluminescence values (relative light units, RLU) were normalized to vehicle controls; data represent averages of three independent replicates. Group means (± SE) and cosinor curves are plotted as a function of time on plate for each treatment condition.

Microarray analyses of exosomal miRNA cargo showed that 62 miRNAs were differentially expressed between the DS and NS conditions, based on early morning time points DS1 and NS5 matched for time of day (Figure 1). Of these, 10 miRNAs achieved statistical significance (*p* ≤ 0.001). See Figure 2 (panel A) for a heatmap with clustering, where *n* = 5 in each condition met microarray experiment quality control criteria. Predictions used in silico tools, revealing 2976 putative target genes, which were assessed with gene ontology and the Kyoto Encyclopedia of Genes and Genomes (KEGG) database; see Tables S1–S3.

Among the top 10 differentially expressed miRNAs that reached statistical significance (Figure 2A), one was selected as proof of concept. We selected hsa-mir-3614-5p, as it showed rhythmic expression with the clearest difference between the DS and NS conditions (Figure 2A). This particular miRNA regulates circadian clock-related genes, with downstream biological KEGG pathways that include insulin secretion and circadian entrainment. Specific miRNA hsa-mir-3614-5p scramble, agomir, and antagomir were transfected into plasma-derived exosomes from subjects in the DS and NS conditions, and subsequently added into cultures of naïve differentiated human adipocytes. The expression of the core clock gene, *BMAL1*, as determined with qRT-PCR, is shown in Figure 2 (panel B, top row left). *BMAL1* expression was significantly lower in the naïve adipocytes treated with exosomes from the NS condition (NS-scramble) as compared to the DS condition (DS-scramble). Treatment with exosomes from the DS condition after transfection of the miRNA hsa-mir-3614-5p agomir reversed the DS effect to become similar to NS-scramble, while treatment with exosomes from the NS condition after transfection of the miRNA hsa-mir-3614-5p antagomir elicited *BMAL1* expression changes similar to DS-scramble. 

Transfection of Bmal1-dLuc U2OS osteosarcoma cells containing a luciferase reporter driven by the Bmal1 promoter [43] with miRNA hsa-mir-3614-5p scramble, agomir, or antagomir elicited dissociated effects (Figure 2B, top row right). The luciferase reporter assay displayed statistically significant 24-h rhythm amplitude (*p* < 0.001) in each case (Figure 2C); however, substantial dampening of the amplitude occurred in the DS-agomir and NS-scramble treatment conditions, but not the DS-scramble and NS-antagomir treatment conditions. These results corroborate a role of exosomal miRNAs, such as hsa-mir-3614-5p, in peripheral clock regulation during experimentally induced circadian misalignment, as further illustrated by the effects on other relevant circadian genes (Figure 2B).

### 2.3. Effects of Exosomes on Transcriptome in Adipocytes Cells

**Figure 3:**
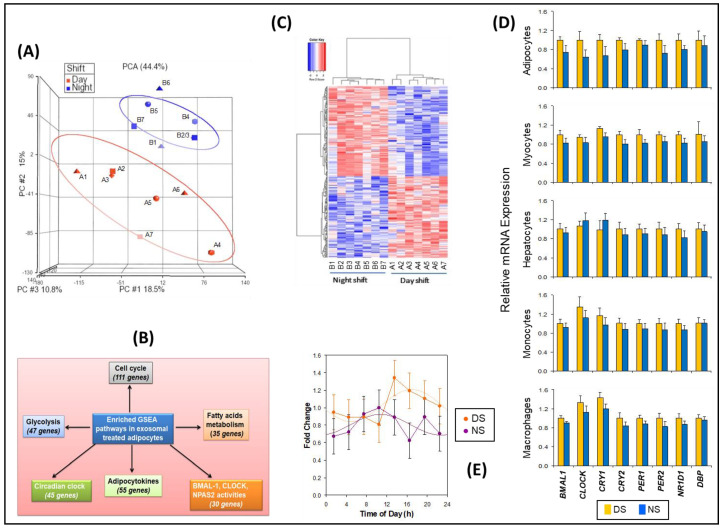
Transcriptomic analyses of naïve differentiated human adipocytes treated with exosomes from the simulated DS and NS conditions, matched for time of day. (**A**) PCA of microarray data. Labels A1–A7 represent individuals in the DS condition; labels B1–B7 represent individuals in the NS condition. Note that B3 overlaps with B2. (**B**) Gene set enrichment analyses (GSEA), confirming induction of broad groups of metabolic and circadian response genes by exosomes from the NS condition. (**C**) Heatmap with clustering of gene expression patterns in human adipocyte cultures treated with exosomes derived from samples of subjects in the DS and NS conditions, using early morning time points DS1 and NS5 matched for time of day (see Figure 1). Each column corresponds to a different subject exposed to either the DS condition or the NS condition. Labels A1–A7 represent individuals in the DS condition; labels B1–B7 represent individuals in the NS condition. (**D**) Relative mRNA expression (means ± SE) as determined with qRT-PCR, for selected circadian clock genes in differentiated human adipocytes, skeletal myocytes, hepatocytes, monocytes, and macrophages after treatment with exosomes from the DS and NS conditions. (**E**) *BMAL1* (*ARNTL*) gene expression of 24-h oscillations in luciferase reporter assay for the eight time points in the DS and NS conditions. Assays were performed in triplicate. Data were expressed as fold change, normalized to fold change in the corresponding β-actin (*ACTB*) RNA level. Results are shown as means and SE (dots and error bars) and cosinor fits (curves).

Treatment of naïve differentiated human adipocytes in vitro with plasma exosomes from samples collected during the constant routine following the DS and NS conditions, using early morning time points DS1 and NS5 matched for time of day (Figure 1), allowed for identification of peripheral gene targets affected by exosome cargo changes in the context of simulated NS work. Genome-wide mRNA expression analysis followed by principal component analysis (PCA) revealed consistent group separation (Figure 3A) with induction of broad groups of metabolic and circadian response genes in the NS condition. We identified multiple genes for each group that were either upregulated or downregulated in the NS condition as compared to the DS condition (Figure 3B), showing that these genes were both induced and decreased during NS. A total of 264 such genes were identified in the NS condition relative to the DS condition (*p* ≤ 0.009), which included circadian clock genes and circadian-regulated transcription factors (Figures S8–S10; Tables S4–S6). 

GSEA retrieved 23,001 native features, and 16,232 genes were identified after collapsing the features into gene symbols. Positive enrichment scores showed 656 out of 917 gene sets that were upregulated for the DS condition and 261 out of 917 gene sets that were upregulated for the NS condition. A heatmap for the top 50 differentially expressed genes between the DS and NS condition, a ranked gene list correlation profile, and a Butterfly plot (Figure S11) showed the positive (gain, red) and negative (lose, blue) correlation between gene rank and the ranking metric score. We identified specific pathways that were prominently detected, including circadian clock, cell cycle, glycolysis, fatty acid metabolism, and adipocytokines, as well as *BMAL1*, *CLOCK*, and *NAPS2* activity pathways (Figure 3C). Enrichment score analysis of the gene sets containing those genes showed increased expression levels in the NS condition (Figures S12–S14), further corroborating these findings. 

Based on the inferential effects of exosomes on circadian pathways and relevant downstream targets, the differential effects of exosomes from the DS and NS conditions on the expression of eight representative clock genes were investigated in several types of naïve peripheral cells: adipocytes, skeletal myocytes, hepatocytes, monocytes, and macrophages. Exosomes from the NS condition widely reduced the expression of these clock genes (Figure 3D). Although our sample size was too small to demonstrate statistical significance of this effect for the individual cell types and target genes, reduced expression occurred in 38 out of 40 cases, which is highly unlikely to be a random result (*p* < 0.001). Thus, exosomes in the circulation may alter peripheral clock expression in a large group of metabolically active cell targets. 

Since circadian misalignment alters peripheral metabolic functions [44] and exposure to 3 days of simulated NS work altered miRNA exosome cargo, the latter may communicate NS-induced circadian misalignment to target organs involved in metabolic homeostasis. In support of this hypothesis, after treatment with exosomes from the DS conditions (all eight time points) the acrophase (peak) of the 24-h rhythm in *BMAL1* expression in differentiated human adipocytes was at 16:56 ± 82 min—as compared to treatment with exosomes from the NS condition (all eight time points), after which the *BMAL1* acrophase was at 10:46 ± 109 min. Thus, exosomes from the NS condition signaled a phase difference of 6.2 h relative to the DS condition (Figure 3E), which was statistically significant (t_12_ = 2.7, *p* = 0.019). 

### 2.4. Effects of Exosomes on Insulin Sensitivity

HOMA-IR values derived from plasma glucose and insulin levels were significantly increased in the NS condition (time point NS5, mean ± SE: 3.58 ± 0.35) compared to the DS condition (time point DS1, mean ± SE: 2.24 ± 0.15) controlling for time of day (*p* = 0.004). Thus, consistent with earlier findings in mice and humans [45,46], the simulated NS paradigm elicited a reduction in systemic insulin sensitivity. 

**Figure 4:**
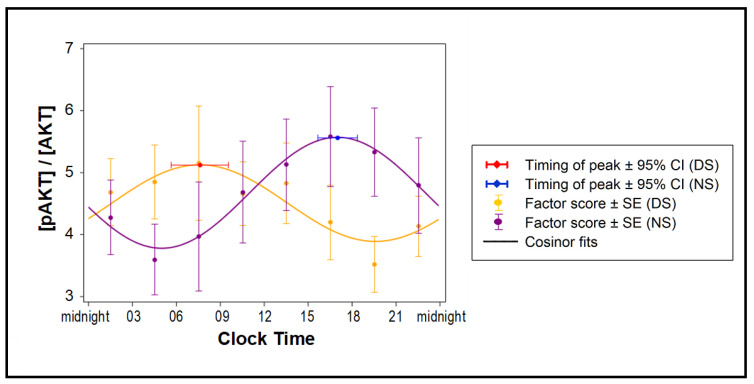
Effect of exosomes on insulin sensitivity of naïve human adipocytes. Changes in the 24-hour average of phosphorylated AKT (pAKT), expressed as fraction of total AKT, for adipocytes treated with exosomes collected every 3 h during the 24 h constant routine following 3 days of simulated DS or NS (*n* = 7 per condition) are shown as means and SE (dots and error bars) and cosinor fits (curves).

Samples from all eight time points of all subjects in the DS and NS conditions (Figure 1) were incubated with naïve differentiated human adipocytes for 24 h. Exogenous insulin was added for the last 30 min, followed by western blots to assess total and phosphorylated Akt. Exosomes from the NS condition markedly shifted Akt phosphorylation responses to insulin when compared to the DS condition (Figure 4). The acrophase of the 24-h rhythm was at 07:35 ± 59 min in the DS condition and at 17:00 ± 41 min in the NS condition, constituting a significant difference of 9.4 h (t_12_ = 7.9, *p* < 0.001). Thus, simulated NS led to an almost complete reversal of the 24-h rhythm of pAKT/AKT insulin responses, along with reduced systemic insulin sensitivity. 

### 2.5. Effects of Exosomes on BMAL1 Binding Sites

ChIP-Seq and bioinformatics were applied to generate genome-wide profiles of *BMAL1* target genes that would be differentially affected by DS and NS exosome cargo. These profiles uncovered 42,700 *BMAL1* binding sites in DS and only 5534 binding sites in NS, with 2481 overlapping between the two conditions (FDR < 0.01). The DAVID Bioinformatics Database for associations with particular gene ontology terms was used to establish a systematic classification of the *BMAL1* target genes (Table S7), which revealed that *BMAL1* target genes are related to rhythmic processes, metabolic pathways, transcription as a biological process, DNA binding or transcription as a molecular function, and nucleus as a cellular component. 

Genome binding sites were identified in 8.46% promoter and 13.10% intronic regions in the NS condition, as compared to 8.19% and 12.90% in the DS condition (Figures S15–S17). *BMAL1* binding sites included 597 genes involved in metabolic processes identified in the NS versus DS conditions, at early morning time points NS5 versus DS1 matched for time of day (Figure S18A; Table S8). Circadian clock and *BMAL-CLOCK-NPAS2* activated circadian gene expression were among the pathways identified (Figure S18B). Furthermore, two highly relevant KEGG pathways were identified, namely PI3K-Akt Signaling and Insulin Resistance (Table S8), which were also identified based on miRNA target genes (Table S2). 

Based on a PubMed search (https://www.ncbi.nlm.nih.gov/pubmed), 57 circadian clock genes and circadian-regulated transcription factors were identified. Considering that circadian regulation operates primarily at the transcriptional level [47], the identified genes were used to perform data mining analyses for circadian clock and circadian-regulated transcription factors genes, yielding a range of potential targets for future exploration of potential interventions (Tables S9–S12). 

## 3. Discussion

The disruption of biological rhythms has been hypothesized to underlie the increase in chronic disease risk in NS workers [1,48]. Epidemiologic evidence and laboratory studies support an association between NS work and metabolic disorders, particularly type 2 diabetes mellitus [3,49,50]. Circadian rhythms play a major role in the homeostatic regulation of metabolism, as demonstrated in both rodents [51,52] and humans [53,54]. In individuals working NS schedules or rotating shifts, as well as in rodent models of circadian arrhythmia, the disruption of the circadian cycle is strongly associated with metabolic imbalance [55,56]. Evidence suggests that misalignment of peripheral oscillators may underlie metabolic disruption [4].

Indeed, the circadian clocks control many biological processes, ranging from molecular and biochemical pathways to physiological and behavioral rhythms, and disruption of daily rhythms leads to adverse health consequences [1]. To maintain approximate 24-hour cycles at the molecular level, clocks must be regulated at several stages to keep the correct period, phase, and amplitude of the rhythms of thousands of proteins that generate the wide range of rhythmic biological processes [57]. Many levels of regulation are important for the proper functioning of the circadian clock, including transcriptional, post-transcriptional, and post-translational mechanisms [57]. Recognition of the role of exosomes in both physiological and pathological conditions significantly increased during the last a few years. Since exosomes are secreted by virtually all cells, they can play a role of mediators of cell-cell communication, cellular differentiation, immunity, and inflammation, and as multi-molecular messengers acting in both autocrine and paracrine ways modifying the activity and/or phenotype of recipient cells [58,59].

In the present laboratory study of simulated shift work, we provided evidence that exosome cargo under NS conditions reflects altered circadian synchrony and exerts changes in circadian clock function (while exosome concentration itself was not affected; Figure S4). Furthermore, we showed that hsa-mir-3614-5p is a functionally relevant mediator of such NS-induced misalignment, along with downstream adverse effects on cellular metabolic function. It has been found that adipose tissue communicates systemically with other organs (brain, liver, skeletal muscle), and also locally with other cells (pre-adipocytes, endothelial cells, and monocytes/macrophages) through secreted products [60,61,62], suggesting that adipose tissue may play a central role in peripheral clock misalignment and the associated metabolic consequences. 

Furthermore, circadian rhythms of peripheral metabolism may be partly controlled via transcriptional regulation by peripheral clock genes. Thus, disturbances of peripheral metabolism could be brought about by disruption of circadian rhythms and their effects on enzyme activity [63]. It is possible, therefore, that exosomes secreted from visceral adipose tissue, skeletal muscle, and hepatocytes, which can transfer functional proteins and RNA species that regulate the metabolic function of both remote tissues and adjacent cells [34,35,64,65,66,67,68] may be messengers of circadian misalignment [69,70]. 

It has been indicated that cells from different organs release exosomes and induce physiological changes in recipient cells upon interaction. Exosomes released from adipocytes in obesity have been proposed to be involved in adipocyte/macrophage cross-talk and to affect insulin signaling [71,72]. Furthermore, NS work-induced alterations in food intake [73] and attendant changes in the microbiome [74,75,76] may affect gut permeability, which could lead to release of exosomes into the circulation to propagate peripheral clock misalignment [46] and consequently affect insulin sensitivity [77]. 

It has been reported that exosomes can be released by cells into the circulation and bodily fluids, and display different protein and RNA contents in healthy subjects versus patients with various diseases, which can be measured as potential biomarkers [78,79,80,81,82]. Exosomes in the circulation are also promising candidate biomarkers of circadian misalignment, due to their unique structural stability and their ability to provide protection for a wide range of cargo including mRNAs, miRNAs and a variety of other, less well-investigated biomolecules such as proteins, enzymes, molecular chaperones, and signaling molecules [26,27]. These same properties make exosomes a potential therapeutic tool for treatment of NS work-induced metabolic disturbance [83]. 

Exosomes show promise for clinical applications both as biomarkers and as therapeutic delivery vehicles. Using exosomes as biomarkers would involve detecting the subset of exosomes from tissue of interest in serum or other biofluids. An advantage of using exosome-derived miRNAs as biomarkers is that they are protected from degradation by RNases and thus are quite stable [26,27]. Furthermore, the vesicles display on their surface the antigenic markers of the cells from which they were derived, allowing enrichment of vesicles from a particular tissue source [84,85]. For therapeutic applications, exosomes could be used to deliver not only miRNAs but also proteins and drugs. Exosome-based therapy is a novel option in regenerative medicine and advanced treatments, and a better understanding of the molecular and cellular processes regulating exosome biogenesis is expected to increase technological advances and potential clinical applications [86]. 

We showed that HOMA-IR values in plasma from subjects in the NS and DS conditions, matched for time of day, were increased after simulated NS work, indicating altered insulin sensitivity. Similar to our results, reduction of insulin signaling in human adipose tissue analyzed by pAkt/Akt ratio has been reported for subjects with insufficient sleep [87]. In our experimental paradigm, where sleep loss was minimized by inclusion of an added, prophylactic nap immediately before the NS condition [88], the observed pAkt/Akt ratio effect was attributable specifically to the reversal of behavioral cycles during simulated NS work prior to the 24-h constant routine when the blood samples were taken [4]. Akt phosphorylation is a crucial step of the PI3K pathway that mediates most of the metabolic actions of insulin, where the effect of insulin via its receptor is determined by the density of pAkt relative to total Akt. An increase in the pAkt/Akt ratio indicates a greater cellular response to insulin. Using high-throughput techniques combined with bioinformatics, we identified the PI3K pathway as highly influenced by the NS condition. Taken together, our data suggest that differentially expressed miRNAs in exosomes in the NS condition may have played a role in altering insulin sensitivity.

The expression of key genes related to circadian rhythmicity—*BMAL1*, *CLOCK*, *CRY1*, *CRY2*, *PER1*, *PER2*, *NR1D1*, and *DBP*—was diminished in the NS condition compared to DS condition. At the molecular level, circadian oscillators consist of a network of genes that, through transcriptional–translational feedback loops, generates tissue-specific circadian rhythmicity in transcription and translation [89,90]. *BMAL1* is a core orchestrator of the molecular clock [11]. It forms a heterodimeric partnership with *CLOCK* and binds to E-box sites located across the genome, thereby inducing rhythmic expression in a multitude of clock-controlled genes [12,91,92]. Based on ChIP-Seq analyses and mRNA microarrays combined with bioinformatics, we generated genome-wide profiles of *BMAL1* target genes. In line with the findings of others [90,93,94], we found that a significant proportion of *BMAL1* targets include genes that encode central regulators of metabolic processes. As such, *BMAL1* may be an important mediator of the effects of NS work on peripheral clock misalignment and insulin resistance. 

To further identify peripheral gene targets affected by exosome cargo changes in the context of simulated NS work, we treated naïve differentiated human adipocytes in vitro with plasma exosomes from samples taken in the NS and DS conditions. Genome-wide mRNA expression analysis pointed to specific regulatory pathways, including circadian clock, cell cycle, glycolysis, fatty acid metabolism, and adipocytokines. These results are consistent with the idea that exosome cargo signals circadian misalignment to peripheral clocks and influences metabolic function.

Among the limitations of the present study, we note the relatively small sample size and the uneven ratio of male:female subjects who were exposed to simulated NS work (6:1) or DS work (4:3). Although no significant sex differences have been observed in measures of the central circadian pacemaker in response to three consecutive night shifts [95], our sample was too small to investigate any sex differences. Additionally, our sample consisted of young adults. The effects of aging on circadian disruption are strong [96], and our results may therefore not generalize to older populations. 

From each of the DS and NS conditions, we used only one time point (DS1 and NS5, matched for time of day) for our miRNA and mRNA experiments. Analyzing only one time point does not reveal the full extent of temporal dynamics of the changes associated with the DS and NS conditions. That said, for *BMAL1* gene expression in the luciferase reporter assay of Figure 3 (panel E) and the insulin sensitivity experiment of Figure 4 we do show complete, 24-h time courses. The agomir/antagomir experiments were conducted at the last stages of all experiments, and limited availability of plasma exosomes prevented us from conducting DS-antagomir and NS-agomir experiments, restricting us to demonstrating mechanistic plausibility. We also did not perform insulin sensitivity assays on skeletal muscle tissue due to limited availability of exosome samples. 

We used commercially available human primary culture cells for the in vitro studies. We did not synchronize or desynchronize the cells in advance of experimentation. Since for each cell type, treatments and cultures were processed identically, we can be confident that differences in response to exosomes from the NS or DS conditions reflect the effects of exosomes. Exosomal functional cargo includes not only miRNA, but also proteins, lipids, mRNAs, and DNA [97]. As such, we cannot conclude from our results that only miRNA accounted for the biological effects observed. It would not have been feasible to evaluate each of the other exosomal cargo elements. However, miRNAs are significant players in exosome signaling because their biological activity turns on or off hundreds or thousands of genes at once [84]. Furthermore, miRNAs have been found to target more than 30% of all protein-coding mRNAs. Therefore, most, if not all, biological processes appear to be influenced by miRNAs at least to some degree. We demonstrated that selected miRNAs in plasma exosomes constitute a major vehicle of intercellular communication, through which circadian misalignment is signaled to peripheral clocks and altered cellular insulin sensitivity is induced in metabolically relevant tissues. 

In future studies, we will process samples using hypothesis-driven source assumptions and then use ImagestreamII FACS-based approaches or proteomic analyses of the vesicle membranes to identify putative source(s) of the exosomes, which we will verify using western blots. Additionally, we plan to vary the degree of exposure to the NS condition to examine whether changes in exosome cargo and miRNA perturbations correlate with insulin resistance or signaling at the cellular level in a dose-response manner.

In conclusion, real-world NS work typically involves little or no adaptation of the central circadian pacemaker [98], and misalignment of peripheral oscillators due to shifted behavioral cycles may be responsible for the adverse health consequences of NS [4]. Although further dissection of the contributions to circadian misalignment by prior wake/sleep and/or feeding/fasting cycles is needed to pinpoint the behavioral source of the circadian misalignment [4,77], we showed that circulating exosomes with altered miRNA cargo serve as a major intercellular signaling pathway of circadian misalignment to peripheral oscillators, leading to metabolic disruption. Confirmatory proof of concept involving a specific, differentially regulated miRNA, hsa-mir-3614-5p, lends credence to the important role played by this exosome-based regulatory mechanism. Our findings raise the possibility of developing miRNA-targeted therapeutic approaches to prevent the long-term metabolic complications of shift work. 

## 4. Methods

### 4.1. Human Subjects

Fourteen healthy volunteers (four women, 10 men; ages 25.8 ± 3.2 y) participated in a laboratory study under highly controlled conditions. In the week prior to the laboratory experiment, subjects maintained a regular sleep/wake schedule, which was confirmed by means of wrist actigraphy, sleep diary, and called-in bedtimes and wake-up times. In the week prior to the study, subjects were asked to refrain from napping, alcohol, caffeine, and tobacco use. See Supplementary Methods for further details. 

The study was approved by the Institutional Review Board (IRB) of Washington State University, and all subjects gave written, informed consent. Analysis of de-identified plasma samples taken during the study was additionally approved by the IRB of the University of Chicago, where the samples were processed. 

### 4.2. Laboratory Experiment and Blood Samples

A seven-day in-laboratory experiment (Figure 1) was conducted under highly controlled conditions in the Sleep and Performance Research Center at Washington State University Health Sciences Spokane. The study began with a baseline day with a nighttime sleep opportunity (22:00–06:00). This was followed by assignment to one of two conditions: a simulated DS condition (*n* = 7) or a simulated NS condition (*n* = 7). The simulated DS condition included 3 days with nighttime sleep opportunities (22:00–06:00). The simulated NS condition included a transition nap (14:00–18:00) followed by 3 days with daytime sleep opportunities (10:00–18:00). In both conditions, breakfast, lunch, and dinner were provided after 1.5 h, 7.0 h, and 13.5 h of scheduled wakefulness, respectively. 

The three days of simulated shift work were followed by a 24-h constant routine protocol [5]. During the constant routine, subjects were kept awake (with continuous behavioral monitoring), the environmental conditions were fixed (constant ambient temperature of 21 ± 1 °C, constant light level below 50 lux), subjects maintained a semi-recumbent posture, and food was distributed equally over the 24 h through hourly snacks [99]. After the constant routine, subjects had a recovery day and then went home. Further details on the laboratory experiment can be found elsewhere [4]. 

During the 24-h constant routine, blood was sampled through an intravenous catheter. Blood was collected in Vacutainer tubes coated with K_2_EDTA. Each blood sample was immediately cold-centrifuged (22,000 rpm) at 4 °C for 10 min. Plasma was extracted, aliquoted, and stored at –80 °C. Selected samples collected at 3-h intervals (Figure 1) were sent to the University of Chicago for exosome-related investigation. 

### 4.3. Overview of Exosome Investigation

**Figure 5:**
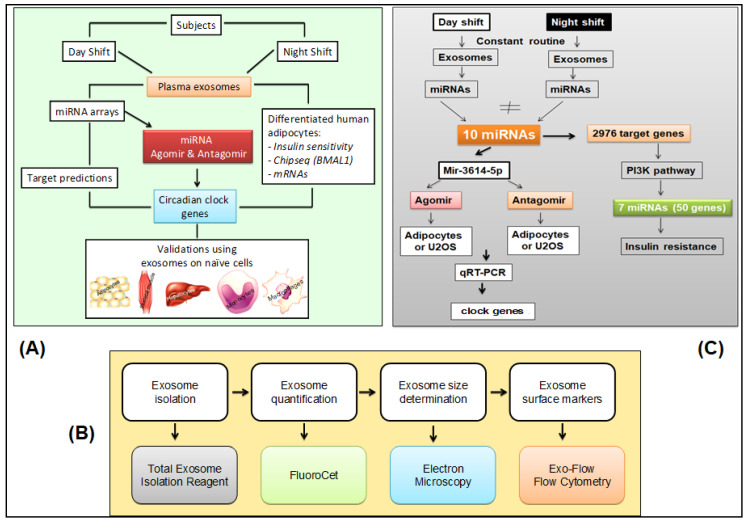
Experimental approaches. (**A**) Schematic of the overall experimental approach. (**B**) Pipeline for isolation, characterization, and quantification of plasma-derived exosomes [25,78]. (**C**) Schematic of miRNA agomir and antagomir selection and processing in cultured human adipocytes.

The overall approach to the investigation of exosomes and their effects in this study is schematically depicted in Figure 5 (panel A). Details of methods and techniques are presented below and in the Supplementary Methods. All assays involving exosomes were performed blind to study condition.

Exosomes were isolated from the blood samples taken during the 24-h constant routine (Figure 1). The plasma exosome isolation and characterization pipeline from blood samples drawn during the 24-h constant routine is shown in Figure 5 (panel B). Negative stain electron microscopy was used to examine exosome morphology. Flow cytometry was used to detect cell components. Exosomes labeled with Exo-Red (for RNAs), Exo-Green (for proteins), or PKH67 (for lipids) were added to naïve differentiated human cell lines in vitro to investigate the incorporation of exosome contents into these cells. DAPI staining was used to reveal co-localization with cell nuclei.

The pipeline for characterization of miRNAs derived from plasma exosomes in blood samples drawn during constant routine is shown in Figure 5 (panel C). Exosomal miRNA cargo was investigated with microarrays. Online databases were used for in silico prediction of putative target genes. Effects on gene expression were investigated with miRNA scramble, agomir, and antagomir transfected into plasma-derived exosomes, which were subsequently added into cultures of naïve differentiated human adipocytes. Gene expression was determined with quantitative reverse transcription polymerase chain reaction (qRT-PCR). Bmal1-dLuc U2OS human osteosarcoma cells containing a luciferase reporter driven by the Bmal1 promoter (U2OS cells) [43] were transfected with miRNA scramble, agomir, and antagomir and used as a reporter assay to investigate 24-h rhythmicity.

Genome-wide mRNA expression was analyzed with microarrays. Gene set enrichment analyses (GSEA) were conducted to determine candidate regulatory pathways. Normalized Absolute Enrichment analysis was used to detect altered gene expression levels. Naïve peripheral cells—adipocytes, skeletal myocytes, hepatocytes, monocytes, and macrophages—were used to investigate the differential effects of exosomes from the DS and NS conditions on the expression of clock genes. 

Systemic insulin sensitivity was assessed using HOMA-IR values derived from plasma glucose and insulin. Akt (protein kinase B) phosphorylation responses to insulin were assessed with western blots [78] to assess total and phosphorylated Akt in exosomes incubated with naïve differentiated human adipocytes for 24 h, with exogenous insulin added for the last 30 min.

Chromatin immunoprecipitation-based sequencing (ChIP-Seq) and bioinformatics approaches were applied to generate genome-wide profiles of target genes of Bmal1 transcriptional activity. Online databases were used to establish a systematic classification of target genes and identify gene binding sites and pathways. Data mining analyses were performed to predict exosome-induced alterations in transcriptional binding in order to identify potential gene targets for intervention.

### 4.4. Glucose and Insulin Assays

Plasma glucose levels were measured in samples from the DS and NS conditions, using time points DS1 and NS5 matched for time of day (Figure 1), by means of glucose assay kit II according to the manufacturer’s instructions (BioVision, Mountain View, CA, USA). Plasma samples were mixed with glucose reaction mixture for 30 min [100,101]. Glucose consumption was calculated from a standard curve. The absorbance was measured using a spectrophotometer at a wavelength of 450 nm or 570 nm. 

Plasma insulin levels were measured using a commercially available human insulin kit (ALPCO, Salem, NH, USA). This method has a detection level of 0.399 μIU/ml and exhibits linear behavior up to 200 μIU/ml, with intra-assay and inter-assay coefficients of variability of 4.86 μIU/ml and 5.10 μIU/ml, respectively. Insulin resistance was assessed using the homeostasis model assessment (HOMA) equation (fasting insulin × fasting glucose/22.5) [45,102,103]. 

### 4.5. Exosome Isolation, Quantification, and Size Determination 

The plasma exosome isolation and characterization pipeline from blood samples drawn during the 24-h constant routine was performed as previously described [15]. To determine unique markers specific to exosomes of purified samples, different exosome markers were used including tetraspanins (CD9, CD63, CD81; exosome formation and secretion), targeting/adhesion (CD31; exosome maturation and target cell binding), and membrane transport (Rab5b; exosome biogenesis, secretion and cell fusion), as shown in Figure S2.

Isolation and characterization of exosomes were performed according to published guidelines [25,104]. Plasma exosomes were isolated using Total Exosome Isolation Reagent (TEIR) according to the manufacturer’s protocol (Life Technologies, Carlsbad, CA, USA) [25,105,106]. Briefly, plasma was centrifuged at 2000× *g* for 22 min to remove cell debris, followed by a second centrifugation at 10,000× *g* for 22 min. See Supplementary Methods for further details.

Exosomes were quantified using enzymatic fluorescent assay (FluoroCet # FCET96A quantitation kit; System Biosciences, Mountain View, CA, USA) according to the manufacturer’s protocol. For all experiments reported here, a quantity of 15 million nanoparticles per sample was used. See Supplementary Methods for further details.

The plasma exosome size distribution was evaluated using electron microscopy, model Tecnai F30, at 300 KV (FEI Company, Hillsoro, OR, USA). Exosomes were placed on Formvar-carbon coated electron microscopy grids, and allowed to stand for 5–10 min for exosome adsorption. Grids with adherent exosomes were transferred to three 25 μL drops of Dulbecco’s phosphate-buffered saline (DPBS) for washing, fixed with 2% paraformaldehyde in DPBS for 7 min, then incubated with 25 μL drops of 2% uranyl acetate and examined by electron microscopy. The size distribution of exosomes was assessed and quantified as previously described [25]. 

### 4.6. Exosome Markers

Purified exosomes were incubated with flow cytometry (Exo-Flow kits; System Biosciences, Mountain View, CA) and analyzed using FACS analysis (FACSCalibur; BD Biosciences, San Jose, CA, USA). Different exosome markers were used based on their functions: tetraspanins (#EXOFLOW150A-1; CD9, CD63 and CD81), targeting/adhesion (#EXOFLOW200A-1; CD31), and membrane transport and fusion (#EXOFLOW500A-1; Rab5a). Negative controls were carried out in the absence of exosomes. Labeled exosomes markers were analyzed on a flow cytometer (FACSCanto II, FACSCalibur; BD Biosciences, San Jose, CA, USA) using FACSDiva software 2.56 (BD Biosciences, San Jose, CA, USA). Data analyses were performed using FlowJo software (Tree Star, Ashland, OR, USA). 

### 4.7. Cellular Uptake of Exosomes

Purified exosomes were labeled with fluorescent linker Exo-Red (System Biosciences, Mountain View, CA), Exo-Green (System Biosciences, Mountain View, CA, USA), or PKH67-Green (Sigma-Aldrich, St. Louis, MO, USA), and further incubated at 37 °C for 10 min. To remove unbound dyes, samples were filtered through a microspin column G-25 (Sigma-Aldrich, St. Louis, MO, USA). The reactions of all samples were stopped by adding ExoQuick-TC reagent, followed by placing the labeled exosome samples at 4 °C for 40 min and centrifugation for 3 min at 14,500× *g*. The pellets were suspended in 1× PBS, and the labeled exosomes were added to human adipocytes, hepatocytes, and myocytes for 6 h in a cell culture incubator at 37 °C. See Supplementary Methods for further details.

### 4.8. Human Cell Cultures

Human cell models for relevant metabolic organs included adipocytes (adipose derived stem cells from a female subject aged 38 y, # PT-5006; Lonza, Walkersville, MD, USA), myocytes (human skeletal muscle myoblasts derived from a male subject aged 19 y, # CC-2580; Lonza, Walkersville, MD), hepatocytes (HepG2 cells # 85011430 from a male subject aged 15 y; Sigma-Aldrich, St. Louis, MO), and monocytes (human acute monocytic leukemia THP-1 ATCC TIB-202 cells from male subject aged 1 y; ATCC, Manassas, VA, USA). All cells were cultured at 37 °C, 5% CO_2_, and 95% relative humidity. Experimentation occurred during the Gap 2/mitosis (G_2_/M) phase of the cell cycle.

Adipocytes: Human adipose derived stem cells (ADSCs) were cultured in pre-adipocyte basal medium (PT-8202; Lonza, Walkersville, MD, USA) supplemented with 10% FBS (Life Technologies, Carlsbad, CA, USA). Cells were seeded in 6-well plates, 200,000 cells/well, in basal medium PGM-2 (Lonza, Walkersville, MD, USA). After 48 h, cells were differentiated by changing the medium to Bulletkit PGM-2 (PT-9502 and PT-8202) medium containing 10% FBS, 5 μg/mL insulin, 1 μM dexamethasone (DEX), and 0.5 mM 3-isobutyl-1-methylxanthine. The medium was changed every 2 days for 12 days. To confirm differentiation into adipocytes, culture medium was removed on day 12 and cells were washed with PBS. Then, cells were fixed with 4% formaldehyde for 30 min at room temperature. Furthermore, cells were washed twice with PBS and stained with 0.6% (w/v) filtered Oil Red O solution (60% isopropanol, 40% water) for 60 min at room temperature (Sigma-Aldrich, St. Louis, MO, USA). Cells were washed with water to remove unbound dye, and visualized by light microscopy. 

Myocytes: Human skeletal muscle myoblasts were cultured in Skeletal Muscle Cell Growth Medium-2 (# CC-3245; Lonza, Walkersville, MD, USA) supplemented with 10% FBS in 6-well plates, and induced to differentiate when they reached 80% confluency (about every 3 days). Cells were washed twice with PBS. Differentiation medium was prepared by adding 2% horse serum to DMEM-F12 medium (both from Invitrogen, Grand Island, NY, USA) for 7 days. Cells were washed twice with cold PBS, fixed with 4% paraformaldehyde/PBS for 1 h at 4 °C, and permeabilized with 0.3% Triton X-100 for 20 min at room temperature. Cells were incubated overnight at 4 °C with primary antibodies including desmin (Cell Signaling Technology, Danvers, MA, USA) and then incubated with anti-rabbit IgG-FITC secondary antibody (Life Technologies, Carlsbad, CA). Cells were counterstained with 1 μg/ml DAPI (4,6-diamidino-2-phenylindole; Sigma-Aldrich, St. Louis, MO, USA) for 1 min, and then observed with fluorescence microscopy.

Hepatocytes: Liver (HepG2) cells were cultured in 6-well plates using Minimum Essential Medium Eagle (ATCC, Manassas, VA, USA) containing 2 mM glutamine, 1% Non-Essential Amino Acids (NEAA), and 10% FBS for 4 days. 

Monocytes, macrophages: Human acute monocytic leukemia THP-1 (ATCC TIB-202) cells were cultured in RPMI 1640 medium containing 2 mM L-glutamine (Sigma-Aldrich, St. Louis, MO, USA) supplemented with 10% FBS. Cells were treated with phorbol-12-myristate 13-acetate (PMA; Sigma-Aldrich, St. Louis, MO, USA) to a final concentration of 100 nM for 2 days. Morphologically, cells then changed from suspension state (monocytes) into adherent state (macrophages). 

Exosomes are abundantly present in serum. There was thus no need to use serum treatment (e.g., 50% adult horse serum or forskolin) to facilitate synchronization of cell-autonomous circadian clocks [107]. We therefore used serum-free media. 

### 4.9. Insulin Sensitivity Assay in Vitro

Human ADSCs were differentiated in 24-well plates. Medium was replaced from differentiated medium to basal medium, and exosomes from each of the eight samples taken during constant routine in all individual subjects (*n* = 7 in the DS condition and *n* = 7 in the NS condition) were added for 24 h in depleted FBS. The cells were exposed to either 0 nM (control) or 5 nM insulin for 30 min, and cells were lysed in RIPA buffer supplemented with protease and phosphatase inhibitor mixture (Sigma-Aldrich, St. Louis, MO, USA) and vortexed briefly. The supernatants were collected after centrifugation at 15,000× *g* for 15 min at 4 °C. See Supplementary Methods for further details.

Insulin sensitivity analyses after exosome treatment were performed for all eight time points of blood collection in each condition (Figure 1) for all subjects (*n* = 7 per condition). Western blots were performed by subject for a total of 16 samples per blot, for pAKT or AKT at the same time, with normalization to subjects’ own AKT data.

### 4.10. Bmal1-dLuc Reporter Assay

Human Bmal1-dLuc U2OS osteosarcoma cells, generated in Dr. Steve A. Kay’s laboratory as previously described [43], were seeded on 96-well white plates at a density of 3 × 10^4^ cells per well, and incubated in a humidified incubator for 24 h (37 °C, 5% CO_2_) in triplicate. In these cells, a lentivirus system (pLenti6; Invitrogen/Thermo Fisher Scientific, Waltham, MA, USA) was applied to deliver Bmal1-dLuc reporter genes into U2OS cells and to establish stable reporter lines by using blasticidin as a selection marker. 

Exosomes derived from samples taken at time points DS1 or NS5, matched for the time of day (Figure 1), were added for 24 h in the same medium supplemented with depleted FBS (System Biosciences, Mountain View, CA, USA), and then, the cell medium was replaced with 100 µl of luciferase medium and placed in the luminescence module using a Photomultiplier Tube detection system (GloMax-Multi Detection System; Promega, Madison, WI, USA). Plates were sealed with adhesive film (Thermo Fisher Scientific, Waltham, MA, USA). Bioluminescence imaging was performed at 37 °C and recorded every 1 h for 24 h. Total bioluminescence values were normalized to vehicle controls.

### 4.11. Exosomal miRNA Isolation and Microarrays

Total RNAs, including miRNAs, were isolated from exosomes derived from samples taken at early morning time points DS1 or NS5, matched for time of day (Figure 1), using miRNeasy Serum/Plasma Mini Kit columns following the manufacturer’s instructions (Qiagen, Valencia, CA, USA). Briefly, the exosome pellets were solubilized in 700 μL Qiazol. Following washing, the spin columns were dried for 5 min. Total RNA was eluted by adding 14 μL DNAse-RNAse-free water to the membrane of the spin column, and incubating for 1 min before centrifugation at 13,000× *g* for 1 min at room temperature. Total RNAs were quantified on a Nanodrop 2000 (Ambion, Austin, TX, USA) and RNA quality and integrity were determined using the Eukaryote Total RNA Nano 6000 LabChip assay (Agilent Technologies, Santa Clara, CA, USA) on the Agilent 2100 Bioanalyzer. The quality of miRNAs was determined using Agilent Small RNA Kit [25]. The miRNA expression analyses were performed using human miRNA microarray for one-color technique (Agilent Technologies, Santa Clara, CA, USA) consisting of 60-mer DNA probes synthesized in situ that represent 2006 mouse miRNAs and 39 viral miRNAs from the Sanger database (version 21).

Total RNA (100 ng) was labeled and hybridized on a microarray (miRNA complete labeling and hybridization kit) and afterwards scanned using DNA Microarray Scanner (Agilent Technologies, Santa Clara, CA, USA). Total RNA including enriched miRNA was dephosphorylated with calf intestine alkaline phosphatase (Agilent Technologies, Santa Clara, CA, USA), denatured with dimethyl sulfoxide, and labeled with pCp-Cy3 using T4 RNA ligase (Agilent Technologies, Santa Clara, CA). The labeled RNAs were hybridized to custom 8×60K human miRNA microarrays (Agilent Technologies, Santa Clara, CA, USA). After hybridization and washing, the arrays were scanned with an Agilent microarray scanner using high dynamic range settings as specified by the manufacturer (Agilent Technologies, Santa Clara, CA, USA). Microarray results were extracted using Agilent Feature Extraction software (v12.0; Agilent Technologies, Santa Clara, CA, USA). The total gene signal was normalized to the 75th percentile of the signal intensity.

### 4.12. Computational Target Predictions and Functional Annotation

Gene targets for differentially expressed miRNAs were initially computationally predicted using established miRNA target-prediction programs: MicroInspector, miRanda, PicTar, RNA22, RNAhybrid and TargetScan. Provided they were reproducibly detected in at least four of the six prediction programs, the predicted genes of individual miRNAs were uploaded to the online Database for Annotation, Visualization and Integrated Discovery (DAVID 6.7, http://david.abcc.ncifcrf.gov) as well as the Ingenuity Pathway Analysis tool version 2.2.1 (IPA, https://www.qiagenbioinformatics.com) for functional annotation and clustering analysis. 

DAVID software was used to identify the most relevant (overrepresented) biological terms associated with a given gene list. Its cluster tool grouped genes based on their associated gene ontology annotations, and the related terms were clustered into groups with enrichment scores calculated from their EASE Score, the modified Fisher exact *p* value [108]. The web server hosts a continuously updated version of the Kyoto Encyclopedia of Genes and Genomes (KEGG) database release 82.1 (https://www.genome.jp/kegg/kegg1.html), which provided a relevant search module based on KEGG pathway descriptions. 

IPA was used to characterize clinical relevance, and molecular and cellular functions related to the identified genes. A cut-off false discovery rate (FDR) of 0.05 was set to identify differentially expressed genes. The right-tailed Fisher’s exact test was performed in IPA to calculate *p* values determining the probability that each biological function assigned to the data set was due to chance alone. *p* values were corrected for multiple comparisons using the Benjamini-Hochberg method for correcting the FDR.

### 4.13. miRNA Agomir and Antagomir in Vitro

Exosomes derived from samples taken at time points DS1 and NS5, matched for time of day (Figure 1), were transfected with specific miRNA (scrambled control, agomir, or antagomir; Life Technologies, Carlsbad, CA, USA) using the Exo-Fect Exosome Transfection Reagent as described by the manufacturer’s protocol (# EXFT20A-1; System Biosciences, Mountain View, CA, USA) as previously reported [25]. Briefly, 50 μL of purified exosomes (100 μg) were used in each reaction, and the following reagents were added: 10 μL Exo-Fect solution, 20 μL of either 20 pmol agomir miRNA or antagomir miRNA, and 70 μL sterile 1× PBS. The mixtures were incubated at 37 °C in a shaker for 10 min and then immediately placed on ice, and 300 μL of ExoQuick-TC was added to stop the reactions. The samples were then centrifuged at 13,000 rpm for 3 min. The transfected exosome pellet was re-suspended in 300 μL 1 × PBS, and 75 μL of the agomir or antagomir was added to approximately 5 × 10^5^ cells per well in 6-well culture plates, grown in exosome-depleted FBS medium. The transfected exosomes were applied into both human differentiated adipocytes and human Bmal1-dLuc U2OS osteosarcoma cells for 24 h. 

### 4.14. mRNA Microarrays

For a whole-transcriptomic analysis of naïve differentiated human adipocytes in vitro, total RNA was isolated from adipocytes treated for 24 h with exosomes derived from the individual subjects in the DS and NS conditions, using samples from early morning time points DS1 (*n* = 7) and NS5 (*n* = 7) matched for time of day (Figure 1). Briefly, purified total RNAs were processed for labeling using the Low RNA Input Fluorescent Linear Amplification Kit (Agilent Technologies, Santa Clara, CA, USA) and hybridized with whole-genome Agilent microarrays (8 × 60 K). Equal quantities of total RNA (25 ng) were labeled with each reaction and 2 μL (34 pg) of RNA spike-in control. The quality of each cRNA sample was evaluated using Agilent 2100 Bioanalyzer (Agilent Technologies, Santa Clara, CA, USA). Each sample was hybridized to an Agilent oligonucleotide microarray for all of the independent experiments (Agilent Technologies, Santa Clara, CA, USA). The microarray slides were scanned using Agilent dual-laser Microarray Scanner and the digitized images were acquired and processed using Agilent Feature Extraction software (v12.0; Agilent Technologies, Santa Clara, CA, USA). 

Background-subtracted intensities were normalized using the quantile method across all remaining microarray experiments. We first corrected microarray expression intensity background using the norm-exp algorithm with an offset value of 50 [109]. The background-corrected data were normalized between arrays using the cyclic loess method [110]. We applied limma moderated t-tests to detect differentially expressed genes, considering the batch effect caused by different chips as covariates in a linear model. *p* values were adjusted by Benjamini-Hochberg method.

Expression values were computed from the raw data and GSEA (version 3.7) were used to interpret the biological significance of genome mRNA expression data as previously described [111,112]. GSEA, a computational methodology to identify classes of genes that are overexpressed in a large set of genes between two biological states, was run according to default parameters. Probes for the same gene were collapsed into a single gene symbol (identified by its HUGO gene symbol), permutation number was set to 1000, and permutation type was set to “gene sets.” The conventional cut-off value for statistical significance used in GSEA is an FDR of 25%. In order to reduce the likelihood of false positive results, however, an FDR cut-off of 5% was used for enriched gene sets.

### 4.15. qRT-PCR Validation

Total RNA was prepared using the RNeasy Lipid Tissue Mini Kit for adipocytes and RNeasy Mini Kit for other cells (both from Qiagen, Valencia, CA, USA), as described by the manufacturer’s protocol. The OD_260/280_ ratios were between 1.9 and 2.1 for all samples. cDNA was prepared using Reverse Transcription Reagents (Life Technologies, Carlsbad, CA). qRT-PCR analysis was performed for selected mRNAs using the ABI PRISM 7500 System (Applied Biosystems, Foster City, CA). To confirm candidate mRNAs, cDNA was synthesized using the Capacity cDNA Archive Kit (Applied Biosystems, Foster City, CA, USA). The thermal cycling conditions were 95 °C for 10 min, followed by 40 cycles at 94 °C for 15 s, 60 °C for 30 s, and 72 °C for 30 s.

The following primers were used: *BMAL1* (Hs00154147_m1), *CLOCK* (Hs00231857_m1), *CRY1* (Hs00172734_m1), *CRY2* (Hs00323654_m1), *PER1* (Hs01092603_m1), *PER2* (Hs00256143_m1), *NR1D1* (Hs00253876_m1), *DBP* (Hs00609747_m1), and housekeeping gene *ACTB* (Hs01060665_g1). All experiments were performed in triplicate. The cycle number (Ct) values were averaged and the difference between the Ct of the housekeeping gene and the Ct of the gene of interest was calculated to determine the relative expression of the gene of interest using the 2^–ΔΔCt^ method. Results are presented as fold change.

### 4.16. Chromatin Immunoprecipitation Sequencing

Human adipocytes were differentiated in differentiating medium for 12 days in 6-well plates. Human differentiated adipocytes were incubated with exosomes from each of the subjects in the DS and NS conditions, using samples from early morning time points DS1 and NS5 matched for time of day (Figure 1), in depleted FBS for 24 h. Cells were collected, and samples were subjected to chromatin shearing, ChIP for BMAL1 protein, library preparation, and quality control, then multiplex next-generation sequencing on an Illumina HiSeq 2500 (Illumina, San Diego, CA, USA). For chromatin isolation, chromatin was isolated using the ChromaFlash kit (# P-2001; EpiGentek, Farmingdale, NY, USA) and sheared using the EpiSonic 2000 Sonication System (# EQC-2000; EpiGentek, Farmingdale, NY, USA). The total volume of sheared chromatin was measured by fluorescence quantification of chromatin associated DNA. The DNA was checked for fragment size distribution of 100–500 bp using Agilent 2100 Bioanalyzer (Agilent Technologies, Santa Clara, CA, USA). Polyclonal anti-BMAL1 antibody-ChIP Grade (# ab3350; Abcam, Cambridge, MA, USA) was validated using the Pre-Sure ChIP Antibody Validation Kit (# P-2031; EpiGentek, Farmingdale, NY, USA). For ChIP reactions, 2.0 μg of BMAL1 antibody was combined with 6 μL of ChIP assay beads, 70 μL of chromatin solution, and 500 μL of ChIP assay buffer in a 1.5 ml centrifuge tube. Samples were incubated at room temperature for 210 min with continuous rotation. After incubation, the beads were washed and the ChIPed DNA was purified and eluted in 12 μL water. Library preparations were performed with all sample targets as well as input controls using DNA End polishing and adaptor ligation (EpiGentek, Farmingdale, NY, USA). Library amplification was performed with indexed primers and library purification, and DNA purified library was eluted with 12 μL water. Library quality control was verified using Agilent 2100 Bioanalyzer (Agilent Technologies, Santa Clara, CA, USA) and KAPA Library Quantification (Roche Sequencing Solutions, Pleasanton, CA, USA). 

For sequencing, 10 nM samples libraries were subjected to next generation sequencing on an Illumina HiSeq 2500 (Illumina, San Diego, CA, USA). Cells not treated with exosomes were used as negative controls. See Supplementary Methods for further details.

### 4.17. Statistical Analyses

Comparisons between conditions employed unpaired Student t-tests, or a non-parametric equivalent when data were not normally distributed, one-way analysis of variance (ANOVA), or two-tailed pairwise comparisons with Student-Newman-Keuls tests. Linear regression was applied to estimate differences between conditions and reference values. Cosinor analyses to investigate 24-h rhythmicity were performed using non-linear mixed-effects regression [113,114], implemented in SAS version 9.4 (SAS Institute, Cary, NC, USA). Comparisons between conditions for cosinor analysis were based on planned contrasts, implemented as t-tests of the difference in parameter estimates between conditions against zero. Controls for non-specific effects of time awake, implemented by adding time awake as a regression term and testing the regression coefficient against zero using t-test, were performed and found to be non-significant.

## Supplementary Materials

Supplementary materials can be found at https://www.mdpi.com/1422-0067/21/17/6396/s1.

## Statement of Significance

Internal desynchronization between the central pacemaker in the suprachiasmatic nuclei (SCN) and peripheral oscillators may underlie the increased risk of metabolic disorders in night shift workers. We identified an exosomal miRNA signaling mechanism and showed in vitro that it can signal circadian misalignment, alter core clock gene expression in peripheral metabolic tissues, and reduce insulin sensitivity of adipocytes. Our results suggest that exosomal miRNAs may serve as a biomarker for detecting susceptibility to night shift work-induced metabolic disease. In addition, our findings suggest that miRNA-targeted therapeutic approaches could be developed to help prevent the long-term metabolic consequences of night shift work.
