# Supplementary material for: Circulating Exosomal miRNAs Signal Circadian Misalignment to Peripheral Metabolic Tissues
Source: Int J Mol Sci. 2020 Sep 3;21(17):6396. doi: 10.3390/ijms21176396 (PMC7503323; doi:10.3390/ijms21176396)
Supplement: Supplementary file 1 [file ijms-21-06396-s001.zip › Khalyfa et al suppl -7-15-2020 HVD.doc]

**Circulating Exosomal miRNAs Signal**

**Circadian Misalignment to Peripheral Metabolic Tissues**

Abdelnaby Khalyfa, Shobhan Gaddameedhi, Elena Crooks, Chunling Zhang, Yan Li,

Zhuanhong Qiao, Wojciech Trzepizur, Steve A. Kay, Jorge Andrade, Brieann C. Satterfield, Devon A. Hansen, Leila Kheirandish-Gozal, Hans P.A. Van Dongen^*^, David Gozal^*^

^*^ Shared senior authorship

**Supplementary Methods**

**Human subjects**

Subjects were screened to be physically and psychologically healthy with no medical or drug treatments, as verified by physical examination, blood chemistry, urinalysis, and validated questionnaires [1]. Women were not pregnant as assessed by blood-based assay. Subjects reported sleeping between 6 and 10 hours in duration and habitually getting up between 06:00 and 09:00, and were not extreme morning- or evening-types as assessed by validated questionnaire. They showed no evidence of any sleep or circadian disorders as assessed by nocturnal polysomnography and validated questionnaires. They had no history of drug or alcohol abuse, were free of traces of alcohol and drugs as assessed by drug screen and breathalyzer, and did not use tobacco products. They were not involved in shift work within three 3 months of entering the study, and did not travel across time zones within one 1 month of entering the study.

**Exosome isolation**

Following first and second centrifugation, plasma samples were filtered using a 0.22 μm centrifuge tube filter (Sigma-Aldrich, St. Louis, MO). For 120 μl of plasma, 60 μl 1X PBS was added followed by 6 μl of proteinase K (Life Technologies, Carlsbad, CA). After incubation at 37 °C for 12 min, 36 μl of Total Exosome Isolation Reagent (TEIR) buffer was added and incubated immediately at 4 °C for 35 min. This was followed by centrifugation at 10,000 × g at 22 °C for 6 min, and pellets were solubilized in 60 μl of 1X PBS [2-4]. Plasma samples were filtered again using a 0.22 μm centrifuge tube filter (Sigma-Aldrich, St. Louis, MO) to remove any large molecules. Exosomes samples were stored at –20 °C until use.

**Exosomes quantification**

Quantification of absolute exosome counts in plasma of subjects exposed to the day shift (DS; *n*=7) and night shift (NS; *n*=7) conditions was performed using the fluorescent FluoroCet assay by utilizing a standard curve of purified and quantified exosomes. Exosomes were quantified using enzymatic fluorescent assay (FluoroCet #FCET96A quantitation kit; System Biosciences, Mountain View, CA) according to the manufacturer’s protocols. The assay was used to measure the esterase activity of cholesteryl ester transfer protein (CETP), and the standard curve was calibrated to known exosome counts.

Enzyme activity of the samples and standards was determined by incubation at room temperature for 20 min. First, exosomes with serial dilutions were optimized (using 25 μl of stock exosomes). Then, exosome standards and actual exosome samples were transferred to 96-well plates. Fluorescence was measured with a fluorescence plate reader (GloMax 9301 Multi-detection System; Promega, Madison, WI) immediately at excitation (530–570 nm) and emission (590–600 nm).

**Cellular uptake of exosomes**

For the human adipocytes, hepatocytes, and myocytes, confluent cell monolayers were grown on 12 cover slips for 24 h in appropriate medium containing 10% fetal bovine serum (FBS), after which cells were washed with medium containing depleted FBS (System Biosciences, Mountain View, CA). Labeled exosomes were added to cover slips, and cells were fixed with 4% (w/v) para-formaldehyde in 1X PBS for 15 min at room temperature, then washed again with PBS. The cell membranes were permeabilized by incubation with 0.25% (v/v) Triton-X-100 in PBS for 10 min. Exo-Red, Exo-Green and PKH67 colors were monitored for delivery into target cells using a Leica SP5 Tandem Scanner Spectral 2-photon confocal microscope (Leica Microsystems, Buffalo Grove, IL) with a 63× oil-immersion lens. As negative control, Exo-Red, Exo-Green and PKH67 were prepared and added to each cell with all reagents, but no exosomes, to monitor unincorporated dyes. Cell nuclei were visualized by staining with DAPI at a concentration of 1 μg/ml in PBS (Life Technologies, Carlsbad, CA) at room temperature for 5 min [5-7].

**Insulin sensitivity assay *in vitro***

After centrifugation and collection of supernatants, protein concentrations of cell lysates were determined using BCA kit (Life Technologies, Carlsbad, CA). The lysates were diluted with sample buffer, separated on SDS-PAGE gel (4%–20%), and transferred to nitrocellulose membranes (Millipore, Billerica, MA). After transfer, membranes were incubated in blocking buffer – 5% nonfat dry milk in 25 mM Tris, pH 7.4, 3.0 mM KCl, 140 mM NaCl and 0.05% Tween 20 (TBST) – for 1 h at room temperature. Membrane were incubated with phosphorylated Ser473 (pAKT) antibody or with Akt antibody (both from Cell Signaling Technology, Danvers, MA) overnight at 4 °C. Membranes were then washed three times for 10 min each with 25 mM TBST. Membranes were incubated with Anti-rabbit IgG, HRP-linked Antibody (Cell Signaling Technology, Danvers, MA) in blocking buffer with gentle agitation for 1 h at room temperature. After washing three times for 10 min each with TBST, immune-reactive bands of membrane were visualized using an enhanced chemiluminescence detection system (Chemidoc XRS+; Bio-Rad, Hercules, CA). Immuno-reactive bands of membrane were quantified by Image Lab software (Bio-Rad, Hercules, CA) [8-10].

**Chromatin immunoprecipitation sequencing**

The quality of raw sequencing reads was assessed using FastQC v0.11.2 (http://www.bioinformatics.babraham.ac.uk/projects/fastqc). Sequencing reads (single-end 50 bp) were mapped to the UCSC human genome model. Unmapped reads were filtered out by samtools v0.1.19. Putative peaks calling was conducted by running Homer wrapped program “findPeaks” with options “-style factor FDR 0.05” on all ChIP-Seq data against their corresponding input data to remove background signal [11]. The identification and annotation of transcription factor binding sites for the target samples was performed in R statistical software with the Bioconductor package ChIPseeker [12]. Functional enrichment analysis of gene ontology terms was performed with clusterProfiler [12] based on the annotated putative potential transcription factor binding sites [13].

**Supplementary TableS**

**Table S1.** List of significant gene ontology including molecular functions, biological processes, and cellular components, as derived from interrogation of predicted target genes using miRNAs differentially expressed in plasma exosomes in the NS condition compared to the DS condition.

**Table S2.** List of most significant KEGG pathways as derived from interrogation of predicted target genes using miRNAs differentially expressed in plasma exosomes in the NS condition compared to the DS condition.

**Table S3.** List of KEGG pathways identified in 50 common predicted target genes for 7 miRNAs involved in PI3K in plasma exosomes in the NS condition compared to the DS condition.

**Table S4.** List of statistically significant, differentially expressed mRNAs identified by transcriptomic analysis of naïve differentiated human adipocytes following treatment with exosomes from the DS versus NS conditions, based on total RNA.

**Table S5.** Circadian clock genes and circadian-regulated transcription factors identified in mRNA arrays of naïve differentiated human adipocytes following treatment with exosomes from the DS versus NS conditions, based on total RNA.

**Table S6.** Top 10 differentially expressed adipocytes cell gene networks following treatment with exosomes from the DS versus NS conditions, based on total RNA.

**Table S7.** Metabolic genes identified in differentiated human adipocytes treated with exosomes from the DS and NS conditions, using samples from early morning time points DS1 (*n*=4) and NS5 (*n*=4) matched for time of day (see Fig. 1).

**Table S8.** KEGG pathways identified by ChIP-Seq for metabolic genes in differentiated human adipocytes treated with exosomes from the DS and NS conditions, using samples from early morning time points DS1 and NS5 matched for time of day (see Fig. 1).

**Table S9.** Potential list of ChIP-Seq calling peaks identified in differentiated human adipocytes treated with exosomes from the DS condition, using samples from time point DS1 (see Fig. 1).

**Table S10.** Potential list of ChIP-Seq calling peaks identified in differentiated human adipocytes treated with exosomes from the NS condition, using samples from time point NS5 (see Fig. 1).

**Table S11.** Potential list of ChIP-Seq calling peaks identified in differentiated human adipocytes treated with exosomes, as overlapping between the DS and NS conditions, using samples from early morning time points DS1 and NS5 matched for time of day (see Fig. 1).

**Table S12**: Summary of number of ChIP-Seq calling peaks identified in differentiated human adipocytes treated with exosomes from the DS and NS conditions, using samples from early morning time points DS1 and NS5 matched for time of day (see Fig. 1).

**Supplementary Figures**

**Figure S1.** Findings from the metabolomics study^S1^ that led to the present investigation of exosomes. **(A)** Plasma melatonin concentrations and dim light melatonin onset (DLMO) as measured during the 24-h constant routine (see Fig. 1) after three days on a simulated day shift (DS) or night shift (NS) schedule. For the DS condition, the DLMO threshold of 10 pg/ml was crossed at 21:22 ± 30 min; for the NS condition, the threshold was crossed at 22:50 ± 34 min. These data show relatively little difference in the timing of the central circadian pacemaker between the DS and NS conditions. **(B)** Dominant temporal profiles of metabolites measured during the 24-h constant routine, as measured with targeted metabolomics, after three days of simulated DS or NS work. Cosinor analysis (fitted curves) shows a nearly complete reversal (12-h shift) of the metabolite rhythms in the NS condition compared to the DS condition. These data indicate substantial misalignment of circadian rhythms in peripheral oscillators in the gut, liver, and pancreas after simulated NS work. **(C)** Total sleep time (TST) during the baseline period (days 1/2; 22:00–06:00) and on the third day of simulated DS work (days 4/5; 22:00–06:00) or NS work (day 5; 10:00–18:00) as measured with polysomnography (PSG); see Fig. 1. These data show relatively little difference in the amounts of sleep obtained between the DS and NS conditions, indicating that the profound desynchronization between the central pacemaker (panel A) and peripheral oscillators (panel B) in the NS condition was not explained by prior sleep history. Panels A and B adapted from Skene and colleagues^S1^ with permission; panel C based on results shown by Skornyakov and colleagues [14].

**Figure S2.** Expression of prototypic surface markers in exosomes as determined with flow cytometry. For tetraspanins (CD9, CD63, CD81), targeting/adhesion (CD31), and membrane transport and fusion (Rab5b), forward scatter height (FSC-H) is shown (top panels) and expressed as a percentage of maximum (bottom) as plotted against fluorescein isothiocyanate area (FITC-A).

**Figure S3.** Exosome size characterization and distribution. **(A)** Representative electron microscopy image for isolated exosomes. **(B)** Relative exosomes size estimation. Scale bar: 100 nm.

**Figure S4.** Exosome quantification in purified plasma samples. **(A)** Average exosome concentrations in samples from the DS condition. **(B)** Average exosome concentrations in samples from the NS condition. Data are plotted against time point (see Fig. 1).

**Figure S5.** Exosome uptake in human naïve differentiated adipocytes *in vitro*. Representative images are shown for cells stained with DAPI (cell nucleus) in medium to which exosomes labeled with Exo-Red (top panels), Exo-Green (middle panels), or PKH67 (bottom panels) were added (left side) or medium to which only the dyes were added but no exosomes (controls; right side). Overlays show that Exo-Red (RNAs) and Exo-Green (proteins) co-localized with cell nuclei, whereas co-localization for PKH67 (lipids) was restricted to the cell membrane (arrows).

**Figure S6.** Exosome uptake in human naïve differentiated skeletal myocytes *in vitro*. Details same as for Fig. S5.

**Figure S7.** Exosome uptake in human naïve differentiated hepatocytes *in vitro*. Details same as for Fig. S5.

**Figure S8.** Gene ontology of differentially expressed mRNAs in naïve differentiated human adipocytes following treatment with exosomes derived from DS versus NS conditions matched for time of day. **(A)** Cellular components, biological processes, and molecular functions are included. **(B)** Top 25 most significant canonical pathways in differentially expressed genes in the NS condition versus the DS condition.

**Figure S9.** Differentially expressed adipocyte gene network following treatment with exosomes derived from samples taken at time points DS1 or NS5, matched for time of day. A representative example is shown (see Table 6) involving cell cycle, cellular assembly and organization, DNA replication, recombination, and repair processes. Continuous and dotted lines indicate direct and indirect interactions, respectively.

**Figure S10.** Differentially expressed adipocyte gene network following treatment with exosomes derived from samples taken at time points DS1 or NS5, matched for time of day. A representative example is shown (see Table 6) involving cellular movement, hematological system development and function, and immune cell trafficking processes. Continuous and dotted lines indicate direct and indirect interactions, respectively.

**Figure S11.** Results of gene set enrichment analyses (GSEA) for mRNA expression in naïve differentiated human adipocytes treated with exosomes from plasma in the NS condition compared to the DS condition. **(A)** Heatmap cluster of the top 50 most statistically significant, differentially expressed genes. Red color indicates up-regulated and blue color indicates down-regulated. Labels A1–A7 represent individuals in the DS condition; labels B1–B7 represent individuals in the NS condition. **(B)** Ranked gene list correlation profile for the DS and NS conditions. **(C)** Butterfly plot for the first and last 100 genes in the ranked list, showing positive and negative correlation between gene rank and ranking metric score. The bottom portion of the enrichment plot shows the observed correlation between gene rank and the ranking metric score for all genes in the ranked list. The normalized enrichment score (NES) is the primary statistic for examining gene set enrichment results. By normalizing the enrichment score, GSEA accounts for differences in gene set size and in correlations between gene sets and the expression dataset.

**Figure S12.** Enrichment scores for selected pathways identified in GSEA. **(A)** In the NS condition, upregulation was seen for cell cycle processes. **(B–F)** In the NS condition, down-regulation was seen for fatty acid metabolism (B), adipokines (C), glycolysis/gluconeogenesis (D), *BMAL1*-*CLOCK*-*NPAS2* activity (E), and circadian clock genes (F).

**Figure S13.** Heatmaps of relative gene expression for selected pathways identified in GSEA: cell cycle, glycogenesis, and fatty acid metabolism. Red denotes relatively high gene expressions, blue denotes relatively low gene expression. Labels A1–A7 represent individuals in the DS condition; labels B1–B7 represent individuals in the NS condition.

**Figure S14.** Heatmaps of relative gene expression for selected pathways identified in GSEA: adipokines, *BMAL1*, and circadian clock genes. Red denotes relatively high gene expression, blue denotes relatively low gene expression. Labels A1–A7 represent individuals in the DS condition; labels B1–B7 represent individuals in the NS condition.

**Figure S15.** Relative distributions of genome locations of *BMAL1* binding sites, including promoter, 3’-UTR, 5’-UTR, exon, intron, and intergenic regions, in naïve differentiated human adipocytes after treatment with exosomes from the DS and NS conditions.

**Figure S16.** List of the top selected enriched reactome pathways that were detected in *BMAL1* binding sites in the DS and NS conditions.

**Figure S17.** Top selected gene ontology enrichment results for detectable *BMAL1* binding sites in the DS and NS conditions. **(A)** Cellular component (CC). **(B)** Molecular functions (MF). **(C)** Biological processes (BP).

**Figure S18.** Metabolic genes for which *BMAL1* targets were detected in ChIP-Seq analysis. **(A)** Gene ontology for metabolic genes found in Fig. S17 panel C. **(B)** Other selected enriched reactome pathways in the DS and NS conditions.

**References**

1.	Skene, D. J.; Skornyakov, E.; Chowdhury, N. R.; Gajula, R. P.; Middleton, B.; Satterfield, B. C.; Porter, K. I.; Van Dongen, H. P. A.; Gaddameedhi, S., Separation of circadian- and behavior-driven metabolite rhythms in humans provides a window on peripheral oscillators and metabolism. *Proc Natl Acad Sci U S A* **2018,** 115, (30), 7825-7830.

2.	Khalyfa, A.; Kheirandish-Gozal, L.; Bhattacharjee, R.; Khalyfa, A. A.; Gozal, D., Circulating microRNAs as Potential Biomarkers of Endothelial Dysfunction in Obese Children. *Chest* **2016,** 149, (3), 786-800.

3.	Khalyfa, A.; Khalyfa, A. A.; Akbarpour, M.; Connes, P.; Romana, M.; Lapping-Carr, G.; Zhang, C.; Andrade, J.; Gozal, D., Extracellular microvesicle microRNAs in children with sickle cell anaemia with divergent clinical phenotypes. *Br J Haematol* **2016,** 174, (5), 786-98.

4.	Khalyfa, A.; Zhang, C.; Khalyfa, A. A.; Foster, G. E.; Beaudin, A. E.; Andrade, J.; Hanly, P. J.; Poulin, M. J.; Gozal, D., Effect on Intermittent Hypoxia on Plasma Exosomal Micro RNA Signature and Endothelial Function in Healthy Adults. *Sleep* **2016,** 39, (12), 2077-2090.

5.	Tian, T.; Wang, Y.; Wang, H.; Zhu, Z.; Xiao, Z., Visualizing of the cellular uptake and intracellular trafficking of exosomes by live-cell microscopy. *Journal of cellular biochemistry* **2010,** 111, (2), 488-96.

6.	Chen, L.; Xiang, B.; Wang, X.; Xiang, C., Exosomes derived from human menstrual blood-derived stem cells alleviate fulminant hepatic failure. *Stem Cell Res Ther* **2017,** 8, (1), 9.

7.	Khalyfa, A.; Youssefnia, N.; Foster, G. E.; Beaudin, A. E.; Qiao, Z.; Pialoux, V.; Pun, M.; Hanly, P. J.; Kheirandish-Gozal, L.; Poulin, M. J.; Gozal, D., Plasma Exosomes and Improvements in Endothelial Function by Angiotensin 2 Type 1 Receptor or Cyclooxygenase 2 Blockade following Intermittent Hypoxia. *Front Neurol* **2017,** 8, 709.

8.	Eljaafari, A.; Robert, M.; Chehimi, M.; Chanon, S.; Durand, C.; Vial, G.; Bendridi, N.; Madec, A. M.; Disse, E.; Laville, M.; Rieusset, J.; Lefai, E.; Vidal, H.; Pirola, L., Adipose Tissue-Derived Stem Cells From Obese Subjects Contribute to Inflammation and Reduced Insulin Response in Adipocytes Through Differential Regulation of the Th1/Th17 Balance and Monocyte Activation. *Diabetes* **2015,** 64, (7), 2477-88.

9.	Xu, G.; Ji, C.; Song, G.; Zhao, C.; Shi, C.; Song, L.; Chen, L.; Yang, L.; Huang, F.; Pang, L.; Zhang, N.; Zhao, Y.; Guo, X., MiR-26b modulates insulin sensitivity in adipocytes by interrupting the PTEN/PI3K/AKT pathway. *Int J Obes (Lond)* **2015,** 39, (10), 1523-30.

10.	Trzepizur, W.; Khalyfa, A.; Qiao, Z.; Popko, B.; Gozal, D., Integrated stress response activation by sleep fragmentation during late gestation in mice leads to emergence of adverse metabolic phenotype in offspring. *Metabolism* **2017**.

11.	Heinz, S.; Benner, C.; Spann, N.; Bertolino, E.; Lin, Y. C.; Laslo, P.; Cheng, J. X.; Murre, C.; Singh, H.; Glass, C. K., Simple combinations of lineage-determining transcription factors prime cis-regulatory elements required for macrophage and B cell identities. *Mol Cell* **2010,** 38, (4), 576-89.

12.	Yu, G.; Wang, L. G.; He, Q. Y., ChIPseeker: an R/Bioconductor package for ChIP peak annotation, comparison and visualization. *Bioinformatics* **2015,** 31, (14), 2382-3.

13.	Gozal, D.; Gileles-Hillel, A.; Cortese, R.; Li, Y.; Almendros, I.; Qiao, Z.; Khalyfa, A. A.; Andrade, J.; Khalyfa, A., Visceral White Adipose Tissue after Chronic Intermittent and Sustained Hypoxia in Mice. *Am J Respir Cell Mol Biol* **2017,** 56, (4), 477-487.

14.	Skornyakov, E.; Gaddameedhi, S.; Paech, G. M.; Sparrow, A. R.; Satterfield, B. C.; Shattuck, N. L.; Layton, M. E.; Karatsoreos, I.; HPA, V. A. N. D., Cardiac autonomic activity during simulated shift work. *Ind Health* **2019,** 57, (1), 118-132.
